# Genome-Wide Identification of NAP1 and Function Analysis in Moso Bamboo (*Phyllostachys edulis*)

**DOI:** 10.3390/ijms23126491

**Published:** 2022-06-10

**Authors:** Yaxing Zhang, Jun Zhang, Deming Yang, Yandong Jin, Xuqing Liu, Zeyu Zhang, Lianfeng Gu, Hangxiao Zhang

**Affiliations:** 1 College of Forestry, Basic Forestry and Proteomics Research Center, Fujian Agriculture and Forestry University, Fuzhou 350002, China; yaxinzhang0823@gmail.com (Y.Z.); 1200421007@fafu.edu.cn (Y.J.); fafuxuqing@163.com (X.L.); zeyubio0212@gmail.com (Z.Z.); 2 College of Life Science, Basic Forestry and Proteomics Research Center, Fujian Agriculture and Forestry University, Fuzhou 350002, China; jessy_1999@163.com; 3 College of Forestry, Fujian Agriculture and Forestry University, Fuzhou 350002, China; demingyang0721@gmail.com

**Keywords:** *Phyllostachys edulis*, histone chaperone, NAP1, rapid growth, flowering, epigenetics

## Abstract

The nucleosome assembly protein 1 (NAP1) family is the main histone chaperone of histone H2A–H2B. To explore the function of NAP1 family genes in moso bamboo (*Phyllostachys edulis*), characterized by extremely rapid growth and a long flowering cycle, we originally conducted a genome-wide analysis of the PheNAP1 gene. The phylogenetic relationship, gene expression pattern, DNA methylation, and histone modification were analyzed. Eventually, 12 PheNAP1 genes were recognized from the *Phyllostachys edulis* genome, divided into two sorts: the NRP subfamily (four members) and the NAP subfamily (eight members). Highly conserved motifs exist in each subfamily, which are distinct between subfamilies. PheNAP1 was distributed homogeneously on 10 out of 24 chromosomes, and gene duplication contributed significantly to the enhancement of the PheNAP1 gene in the genome. *Cis*-acting element analysis showed that PheNAP1 family genes are involved in light, hormone, and abiotic stress responses and may play an important role in the rapid growth and flowering. PheNAP1 exhibited the highest expression level in fast-growing shoots, indicating it is closely associated with the rapid growth of moso bamboo. Besides, PheNAP1 can rescue the early-flowering phenotype of *nrp1-1 nrp2-2*, and it affected the expression of genes related to the flowering pathway, like *BSU1*, suggesting the vital role that PheNAP1 may take in the flowering process of moso bamboo. In addition, histone modification results showed that PheNAP1 could bind to phosphorylation-, acetylation-, and methylation-modified histones to further regulate gene expression. A sketch appears: that PheNAP1 can accompany histones to regulate fast-growth- and flowering-related genes in moso bamboo. The consequences of this study enrich the understanding of the epigenetic regulation mechanism of bamboo plants and lays a foundation for further studies on the role of the NAP1 gene in *Phyllostachys edulis* and the function of chromatin regulation in forest growth and development.

## 1. Introduction

Histone chaperones can bind histones, mediate the interaction between histone and DNA during nucleosome assembly/disassembly [1,2], and participate in the regulation of the expression of many important plants’ developmental genes, playing a vital role in many biological processes [3]. NAP1 family histone chaperones are the main chaperones of histone H2A–H2B [2]. NAP1 was originally obtained from *Xenopus laevis* and later isolated from human and mouse cells, and it is evolutionally conserved in various species [1]. The first gene encoding NAP1 in plants was reported to *Glycine max* [4], and the NAP1 sequence was later identified in certain plants, including *Nicotiana tabacum* (NtNAP1), *Oryza sativa* (OsNAP1), and *Arabidopsis thaliana* (AtNAP1) [5,6]. Both *Arabidopsis thaliana* and *Oryza sativa* contain 4 distinct subtypes of NAP1, namely *NAP1-1*, *NAP1-2*, *NAP1-3*, and *NAP1-4*, as well as two additional members from the same family, known as NAP1-related proteins (NRPs). NAP1 is involved in many biological processes, including chromatin replication, DNA repair, homologous recombination, root meristem formation, abiotic stress response, and gene-expression regulation. NRPs have a conserved domain similar to NAP1, but they differ from NAP1 in phylogeny and structure [6,7,8].

Studies on NAP1 in *Arabidopsis thaliana* have shown that the loss of either NAP1 or NRP subfamily members will lead to a decrease in the frequency of somatic homologous recombination under normal growth conditions or after gene agent treatment [9]. Simultaneous deletion of the two genes of NRP resulted in a short-root phenotype and sensitivity to gene-agent stress [7]. Genes of the NAP1 subfamily affect the growth of the first pair of leaves in plants and promote cell division and extension, and some members are specifically expressed in roots and pollen grains [10]. In conclusion, the loss of NAP subfamily members and NRP subfamily members will lead to the abnormal growth and development in plants [11], which further indicates that NAP1 family members assume a significant part in plant growth.

*Phyllostachys edulis*, one of the fastest growing plants [12], is a scattered bamboo species of the *Poaceae Barnhart* subfamily, which present as widely distributed and have a high economic, ecological, and social value [13,14]. Due to its unique characteristics of “explosive growth” [15], *Phyllostachys edulis* has been regarded as the plant type with the most potential in the 21st century. At the same time, *Phyllostachys edulis* is a perennial, once-flowering plant. After flowering, it will die.

Research on the growth and genetic improvement of *Phyllostachys edulis* has attracted much attention, but flowering development and epigenetic regulation have always been the focus and the cause of difficulty in the research. Scarcely any histone chaperones have been reported on *Phyllostachys edulis*, despite a number of genetic studies in recent years. Due to NAP1 being the main chaperone of histone H2A–H2B, the main purpose of this study was to identify NAP1 genes in *Phyllostachys edulis* and to explore the underlying epigenetic functions and their roles in flowering. We hypothesis that it may be related to the rapid growth and flowering of *Phyllostachys edulis*.

Here, we provide an overview of the NAP1 gene family for the first time, which will establish a foundation for future functional studies, especially the role of NAP1 protein in chromatin regulation in forest growth and development, and further enrich the understanding of the epigenetic regulation mechanism of bamboo plants. Transcriptome data and RT-qPCR were utilized to analyze the expression of NAP1 in various tissues of *Phyllostachys edulis*, and genetic transformation was used to further elucidate the role of NAP1 in the flowering of *Phyllostachys edulis*.

## 2. Results

### 2.1. Identification of the NAP1 in Phyllostachys edulis

Using NAP1 amino acid sequence of *Arabidopsis thaliana* as the template, BLASTP search was performed on genomic data of *Phyllostachys edulis* and the HMM program analysis showed 12 candidate proteins of NAP1 family were identified, which were named PheNAP1:1 to PheNAP1:12 (Table S1).

### 2.2. Phylogeny, Gene Structural and Conserved Motifs Analysis of NAP1

**Figure 1:**
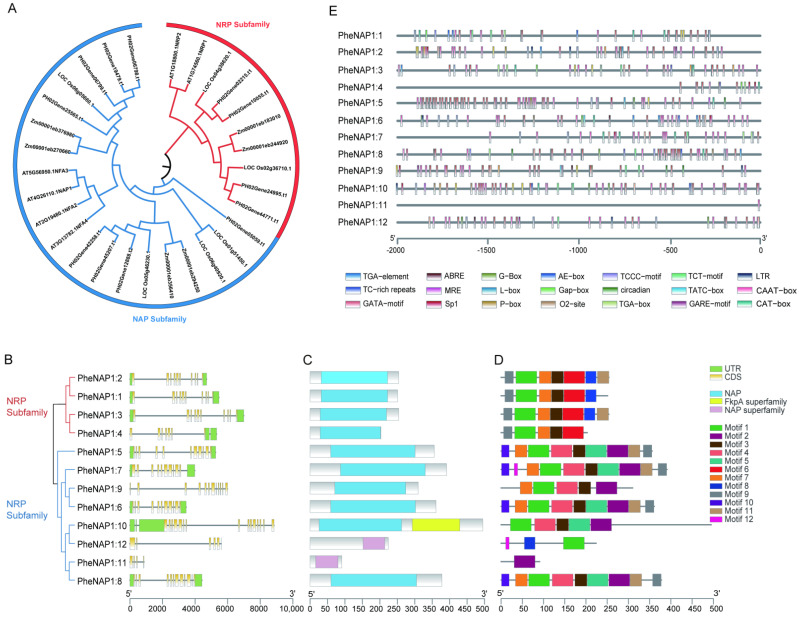
Evolutionary and structural analyses of the NAP1 family in *Phyllostachys edulis*. (**A**) Phylogenetic analysis of NAP1. Phylogenetic trees (neighbor-joining) were constructed using all candidate NAP1 proteins from *Phyllostachys edulis*, *Oryza sativa*, *Zea mays*, and *Arabidopsis thaliana*. The tree is divided into two subfamilies: the NRP subfamily in red, and the NAP subfamily in blue. (**B**) The gene structure of the PheNAP1 gene family, UTR (green rectangle), exons (yellow rectangle), and introns (gray line) are shown along with its sequence length. (**C**) Conserved domain of PheNAP1 family proteins. (**D**) Conserved mods of the PheNAP1 family proteins, with motif 1–motif 12 displayed in differently colored boxes. (**E**) The PheNAP1 genes are shown on the left; the scale at the base indicates the length of the promoter sequence, and the differently colored boxes represent different *cis*-acting elements.

In order to explore the evolutionary relationship and classification of the NAP1 protein, we selected full-length amino acid sequences of the NAP1 protein from *Phyllostachys edulis*, *Oryza sativa*, *Zea mays*, and *Arabidopsis thaliana* to construct a neighbor-joining (NJ) phylogenetic tree (Figure 1A). Based on the classification of *Arabidopsis thaliana* and *Oryza sativa*, the PheNAP1 family can be clustered into two subfamilies: the NAP subfamily (blue) and the NRP subfamily (red). Each of these species contains members of these two subfamilies, suggesting that the two subfamilies exist in monocotyledons and dicotyledons. The PheNAP subfamily exists in 8 members, while the PheNRP subfamily consists of 4 members, including PH02Gene24995.t1 (PheNAP1:1), PH02Gene44771.t1 (PheNAP1:2), PH02Gene02215.t1 (PheNAP1:3), and PH02Gene10055.t1 (PheNAP1:4). *Oryza sativa*, *Zea mays*, and *Arabidopsis thaliana* have four members in the NAP subfamily and two members in NRP subfamily, respectively.

The different structure of the gene has assumed a significant part in the evolution of the species. To investigate the function of PheNAP1 family in *Phyllostachys edulis*, we analyzed its gene structure, conserved domain, and conserved motifs. We observed that members with close phylogenetic relationships share similar genetic structures but also differences, raising the possibility of some degree of functional diversity.

The number of exons in PheNAP1 was mostly 10–12; at the most, 15 (PheNAP1:10), and at the least, only 4 (PheNAP1:11) (Table S1, Figure 1B), and genes with fewer exons may lose some functions in evolution. In addition, a protein domain search was conducted in the NCBI CCD database, and we found 12 candidate protein lengths, ranging from 91 amino acids (PheNAP1:11) to 495 amino acids (PheNAP1:10) (Table S1), including a conserved NAP domain or NAP-superfamily domain, a variable-length N-terminal extension, and a C-terminal tail (Figure 1C).

The PheNAP1 family is basically located in the nucleus, except PheNAP1:9 (in the chloroplasts, mitochondria, and nucleus), PheNAP1:10 (in the chloroplasts), and PheNAP1:12 (in the chloroplasts and nucleus) (Table S1), which may be caused by the distinct localization signal. PheNAP1:10 contains an FkpA superfamily domain (Figure 1C), which may be closely related to its distinct function in the chloroplasts.

We used the MEME online analysis tool to search the conserved motifs in the PheNAP1 family, and the results showed that there were 12 conserved motifs, named Motif 1 through Motif 12 (Figure 1D). Each subfamily contained highly conserved motifs. Among them, the members of the NRP subfamily all contain Motif 1, Motif 3, Motif 6, Motif 7, and Motif 9, while members of the NAP subfamily all contain Motifs 1–4. Many motifs exist in specific subfamilies and may perform specific biological functions, as do Motif 2, Motif 5, and Motif 10 in the NRP subfamily.

### 2.3. Promoter Region Analysis of PheNAP1

In order to investigate the role of *cis*-acting regulation in the growth and development of *Phyllostachys edulis*, the promoter sequence of PheNAP1 was analyzed, and 19 *cis*-acting elements were identified, which are mainly associated with light response, hormone response, and abiotic stress response (Figure 1E). Among them, GAP-box, AE-box, GATA-Motif, G-box, SP1, TCCC motif, CTT motif, L-box, and MRE are light-responsive elements, indicating that PheNAP1 may be involved in plant photomorphogenesis. Circadian is involved in circadian rhythm control in plants and is a biological clock response element. TGA-box and TGA-Element are auxin response elements. Gare-Motif, P-box, TATC-box, and ABRE are involved in gibberellin and abscisic acid reactions, respectively. LTR and TC-rich repeat are related to low-temperature responsiveness and defense and stress responsiveness, respectively. CAT-box is a *cis*-acting regulatory element related to meristem expression. These results indicate that various functions of PheNAP1 will be involved in *Phyllostachys edulis*. It is worth mentioning that PheNAP1:11 is predicted to only have CAAT-box, which implies that it may have a distinct expression pattern.

### 2.4. Chromosome Location, the Ka/Ks, and Synteny Analysis of PheNAP1

**Figure 2:**
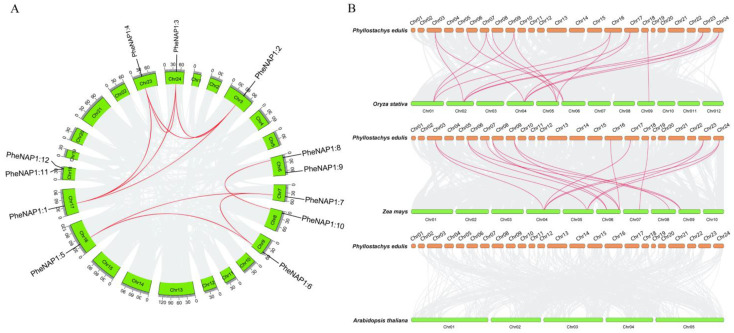
Chromosome location and synteny analysis of NAP1 genes in *Phyllostachys edulis*. (**A**) Synteny analysis of PheNAP1 genes. Red lines indicate duplicated NAP1 gene pairs; gray lines represent the synteny result of the moso bamboo genome. (**B**) Synteny analysis of NAP1 gene in *Phyllostachys edulis* and other plants. The gray lines in the background represent syntonic blocks of *Phyllostachys edulis* with *Oryza sativa*, *Zea mays*, and *Arabidopsis thaliana*, and the red lines represent syntonic NAP1 gene pairs.

All of the PheNAP1 genes were unevenly distributed on 10 chromosomes (Figure 2A and Figure S1). The results showed that two PheNAP1 genes were found on Chr 06 and Chr 18, and only one PheNAP1 gene was found on Chr 03, Chr 07, Chr 08, Chr 09, Chr 16, Chr 17, Chr 23, and Chr 24. The uneven distribution of these genes indicates that there could be genetic differences in the process of evolution.

Among the 12 PheNAP1 genes, a total of 10 pairs of segmental duplicates were identified in *Phyllostachys edulis* (Figure 2A and Table S2). Gene duplication events are especially important in the evolution of gene families and assume a critical part in development and environmental adaptation through the continuous expansion of gene families to acquire new genes. Of the 10 homologous duplicated gene pairs we identified, all homologous gene pairs were shaped by segmental duplication or whole-genome duplication. To further study the repeated pattern of the PheNAP1 gene in the evolution process, we calculated the replacement rate of non-synonymous substitution (Ka) and synonymous substitution (Ks) for each paralogous pair (Table S2). The Ka/Ks proportion went from 0.1415 to 0.3605, and the Ka/Ks ratio of the 10 duplicated PheNAP1 gene pairs were less than 1, demonstrating that these genes underwent a purification selection of evolution. The analysis of 427 moso bamboo individuals, 12 PheNAP1 genes were found, showing low genomic diversity [16]. Only PheNAP1:4 may be affected by structural variants (SVs), and PheNAP1:7 was detected in the long, continuous, heterozygous, SNP-clustered regions of high frequency in Chr.7: 38,000,001–44,200,000.

Furthermore, synteny analysis was performed between *Phyllostachys edulis, Oryza sativa*, and *Zea mays* to explore the evolutionary relationship of NAP1 genes among various species (Figure 2B). The outcomes showed that a sum of 17 orthologous gene pairs were identified in *Oryza sativa* and *Phyllostachys edulis*, which were orthologous with Chr1, Chr2, Chr4, Chr5, Chr6, and Chr9 in *Oryza sativa*. An aggregate of 16 NAP1 orthologous gene pairs was identified in *Zea mays* and *Phyllostachys edulis*. PheNAP1 genes were mainly orthologous with Chr4, Chr5, Chr6, Chr7, Chr8, and Chr9 in *Zea mays*. Although orthologous gene pairs are similar in *Phyllostachys edulis, Oryza sativa*, and *Zea mays*, there are still differences, such as that PheNAP1:7 (Chr7), PheNAP1:5 (Chr16), and PheNAP1:1 (Chr17) have two orthologous pairs in *Oryza sativa*, while the number is only one in *Zea mays*. The results showed that the NAP1 family genes have assumed a significant part in the evolution of various species. It is noteworthy that there was a large orthologous relationship between *Phyllostachys edulis, Oryza sativa*, and *Zea mays*, but there was no such relationship between *Phyllostachys edulis* and *Arabidopsis thaliana*. This might be on the grounds that *Phyllostachys edulis*, *Zea mays*, and *Oryza sativa* are monocotyledons, while *Arabidopsis thaliana* is dicotyledonous, suggesting a closer phylogenetic relationship between monocotyledons.

### 2.5. Expression Profiles of PheNAP1 Genes among Different Tissues

**Figure 3:**
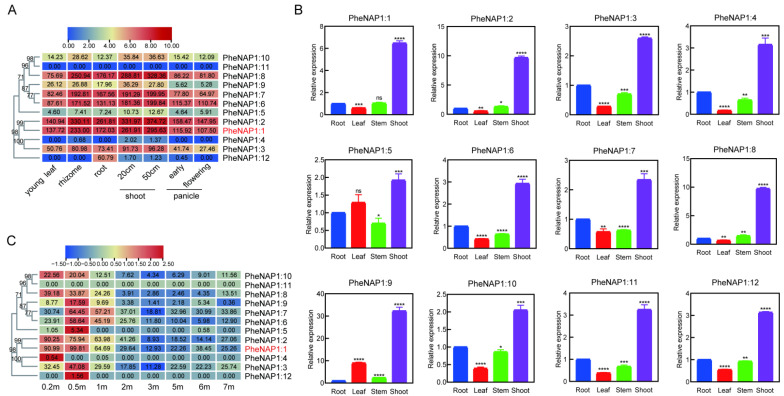
Expression profiles of PheNAP1 family genes in *Phyllostachys edulis*. (**A**) The clustering heatmap shows the expression patterns of PheNAP1 in different tissues (leaf, root, shoot, and panicle) using transcriptome data. (**B**) Expression of PheNAP1 family genes in the root, leaf, stem, and shoot by RT-qPCR analysis. We performed three biological replicates and three technical replicates. Error bars indicate standard deviation. *: *p* < 0.05; **: *p *< 0.01; ***: *p *< 0.001; and ****: *p* < 0.0001. (**C**) The clustering heatmap indicates the expression pattern of *PheNAP1* in fast-growing shoots with heights from 0.2 m to 7 m using transcriptome data.

To concentrate on the expression pattern of the NAP1 gene and its role in the growth and development of *Phyllostachys edulis*, RNA-seq data published by predecessors were utilized for analysis [15]. Gene expression levels at different stages in different tissues (young leaf, rhizome, root, shoot, and panicle) were compared. With the exception of *PheNAP1:11*, *PheNAP1:4*, and *PheNAP1:12*, the other members were detected as being expressed at all stages. From the heatmap, we could see that the expression level of the PheNAP1 members is generally the highest in the shoot, followed by the rhizome, and it is the lowest in the flowering stage (Figure 3A). It is noteworthy that PheNAP1:1 and PheNAP1:2 were found to be constantly highly expressed in all tissues. Further, we checked the expression level of PheNAP1 in the root, stem, leaf, and shoot via RT-qPCR, and we found that all of the PheNAP1 members were expressed in these tissues, and, similar to the above observation, the level in the shoot was significantly higher than that in the root, stem, and leaf (Figure 3B), which indicates that PheNAP1 may be associated with the fast growth of *Phyllostachys edulis*. PheNAP1:9 was the highest, with a relative expression value of 32, followed by PheNAP1:8, PheNAP1:2, and PheNAP1:1 in the sequence, and PheNAP1:5 was the lowest, with a 1.9 relative expression value. To the transcriptome data of the rapidly growing shoots, from 0.2 m to 7 m, showed that PheNAP1 increased from 0.2 m and reached its highest level in 0.5 m shoots, and then it decreased (Figure 3C). The shorter the shoots, the more vigorous the division. These results indicated that the structural and quantitative changes in the chromosomes resulted in the high expression of histone chaperones during nucleosome assembly, which further indicated that NAP1 played a vital role in the rapid growth of moso bamboo. Besides, *PheNAP1:1* and *PheNAP1:2* were found at relatively constant and higher expression levels than other members in different stages and tissues, which indicates that they may have a more important function (Figure 3C). These results indicated that PheNAP1 had a tissue expression specificity with a high expression level in fast-growing parts, such as the meristem.

### 2.6. Functional Study of PheNAP1:1

**Figure 4:**
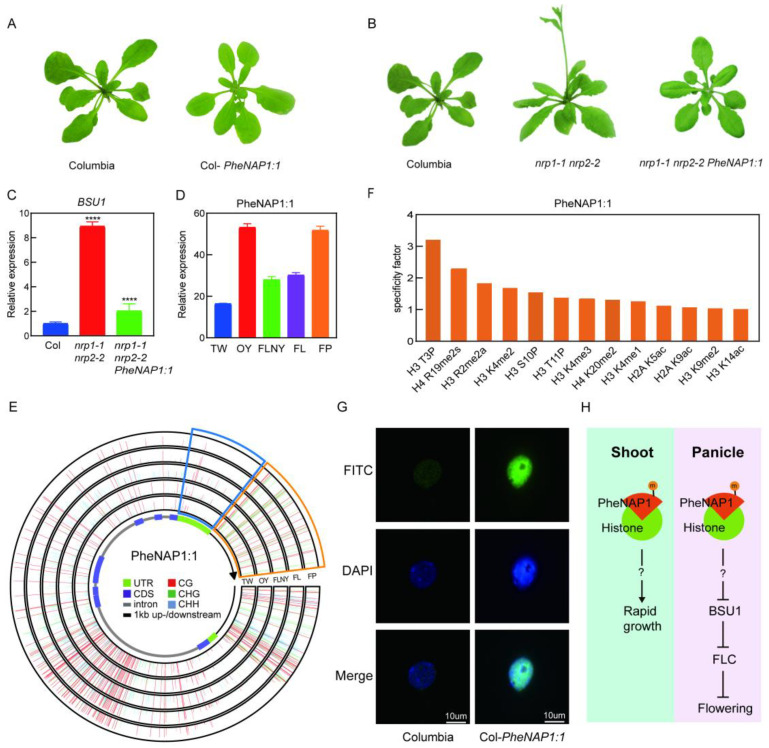
Functional analysis of PheNAP1:1. (**A**) The phenotype of *Arabidopsis thaliana* (Ecotype Columbia) and transgenic plant Col-*PheNAP1:1* grown under long-day conditions. (**B**) Morphological phenotype of Columbia, *nrp1-1 nrp2-2*, and transgenic plant *nrp1-1 nrp2-2 PheNAP1:1* grown under long-day conditions. (**C**) Relative expression of *BSU1* in Columbia, *nrp1-1 nrp2-2*, and *nrp1-1 nrp2-2 PheNAP1:1* measured by RT-qPCR. Error bars represents standard deviation. ****: *p* < 0.0001. *UBQ10* was used as an internal control. (**D**) Expression analysis of *PheNAP1:1* in different tissues according to transcriptome data. TW: leaves of 3-week-old seedlings, OY: leaves of 1-year-old plants, FLNY: leaves of plants that will flower in the next year, FL: leaves of flowering plants, FP: flower florets. (**E**) Comparison of DNA methylation profile of PheNAP1:1 in TW, OY, FLNY, FL, and FP. Wiggle plot showing methylation level in gene body and up-/downstream region of the PheNAP1:1 locus. (**F**) Histone modification analysis of PhNAP1:1. (**G**) Immunofluorescence of PheNAP1:1. FITC: fluorescein isothiocyanate. (**H**) The assumed pathway PheNAP1:1 involved. M: DNA methylation.

The only known structures of the NAP1 family in *Arabidopsis thaliana* are NRP1 and NRP2 [8]. Studies have shown that AtNRP2 may be more effective as a histone chaperone by studying histone dimers and the interaction between AtNRP1 and AtNRP2 [17]. In order to study the function of PheNAP1 in *Phyllostachys edulis*, we used the amino acid sequence of AtNRP2 in *Arabidopsis thaliana* as the template to determine the homology (PheNAP1:1, PH02Gene24995.t1) in *Phyllostachys edulis* by BLASTP. Overexpressed *PheNAP1:1* in wild-type plants (Col-*PheNAP1:1*) showed no special difference compared with the wild-type (Columbia) (Figure 4A).

According to previous studies, the *nrp1-1 nrp2-2* double mutant of *Arabidopsis thaliana* has an early-flowering phenotype compared with the wild type [18], so we speculated that NRPs may be related to some flowering mechanisms. Thus, a similar function of PheNAP1 was supposed in *Phyllostachys edulis*. Interestingly, *PheNAP1:1* transgenic plants in *Arabidopsis thaliana* (*nrp1-1 nrp2-2 PheNAP1:1*) can partially rescue the early-flowering phenotype (Figure 4B), indicating that *PheNAP1:1* may play a vital role in regulating the flowering process.

*nrp1-1 nrp2-2* has an early-flowering phenotype and affects the expression of the genes required for development, such as *BSU1* and *FLC* [18]. *BSU1* is located upstream from *FLC* and inhibits *FLC* expression, which inhibits flowering. *BSU1* was significantly upregulated in *nrp1-1 nrp2-2* (Figure 4C), and the expression level of *BSU1* in the complementary transgenic plant *nrp1-1 nrp2-2 PheNAP1:1* was closer to that of the wild type (Figure 4C), indicating that the expression of NRPs has a negative relationship with *BSU1* and may affect the flowering by regulating the expression of *BSU1*.

It is well-known that most gramineous 1-year-old plants are at maturity and have the ability to flower, whereas bamboo plants rarely flower. Subsequently, transcriptome data of moso bamboo at different growth stages were used to analyze PheNAP1:1 (Figure 4D). PheNAP1:1 was highly expressed in FP (flower florets) and leaves from OY (1-year-old plants), while PheNAP1:1 was relatively low in FLNY (flower in the next year) and FL (flowering plants). It seemed that the higher expression level of PheNAP1:1 corresponded to the more vigorous culms, again suggesting that PheNAP1 may participate in regulating the rapid growth of moso bamboo. The intense expression of PheNAP1:1 in the division of organization and flower material was consistent with that expressed in shoots at different heights, which strengthens the deduction that it would be involved in flowering.

In addition, DNA methylation plays a key role in gene expression. In order to study the relationship between flowering stage and methylation level of PheNAP1:1, the global methylation level was depicted according to different stages (Figure 4E) [19]. It showed that the DNA methylation of PheNAP1:1 changed dramatically during the phase transition, and FL and FP had less frequent methylation, especially in 3’ UTR, than in TW, OY and FLNY. Interestingly, CHH methylation shows a slightly greater frequency in the 3’ downstream of the TE transcription terminal sites (TTS) in FL.

In the context of chromatin, neither DNA methylation nor histone modification exist independently; there is a strong connection between them. Histone post-translational modifications can affect the structure and function of chromatin and intently partake in DNA processes, suggesting that they have a significant part in regulating gene expression. In order to further clarify histone modification in the PheNAP1 gene-expression regulation function, we performed histone modification testing using a purification of PheNAP1:1 protein, and the results showed that PheNAP1:1 could directly bind to the classically modified histones, especially through phosphorylation and methylation, as well as a small amount of acetylation (Figure 4F).

In order to determine PheNAP1:1 localization in cells, we constructed a PEG-Ubi-PheNAP1:1 fusion expression vector (Figure S2). Immunofluorescence was used to analyze its localization, and the results showed that PheNAP1:1 distributed in the euchromatin region of the nucleus, but not in the heterochromatin region, such as in the chromosome center and nucleoli, and it was a nuclear-localization protein (Figure 4G).

In moso bamboo, PheNAP1, as a histone chaperone, was expressed at high levels in the panicle and shoots. It can accompany histones to affect *BSU1* and *FLC* expression, and eventually it regulates the flowering process (Figure 4H). In addition, the constantly high expression of PheNAP1 in the fast-growing stages indicated that it may be related to the rapid growth of moso bamboo. Epigenetic regulation plays a very important part of moso bamboo.

## 3. Discussion

Epigenetics has assumed a significant part in plant growth and development, and histone and histone chaperones are important in the regulation of gene expression, genomic stability, the cell cycle, and biological and abiotic stress [20]. NAP1 is the most important histone chaperone of histone H2A–H2B, and it is involved in chromatin deposition and flowering regulation. Although NAP1 was firstly reported in *Glycine max* [4] and successively identified in *Arabidopsis thaliana*, *Nicotiana tabacum*, and *Oryza sativa* [5,6], it has not been reported in other plants. Here, we conducted a genome-wide analysis of the NAP1 family in *Phyllostachys edulis* and preliminarily explored the potential function of PheNAP1.

An aggregate of 12 PheNAP1 family members was identified and partitioned into two subfamilies, which is consistent with the classification in *Nicotiana tabacum* and *Oryza sativa* [5]. Comparing the composition of the two subfamilies, the number of NRP subfamily members in *Phyllostachys edulis* is four, and that in *Arabidopsis thaliana*, *Oryza sativa*, and *Zea mays* is 2, which might be due to the fact that bamboo is tetraploid [21], while *Arabidopsis thaliana*, *Oryza sativa*, and *Zea mays* are diploid. The consequences of gene structure analysis showed that members of the same subfamily were similar, but there were also differences, demonstrating that genes in the same subfamily may have the same function, but there is also the possibility of functional diversity.

Gene duplication events play a crucial part in gene family evolution and can improve gene structure and enhance functional diversity by expanding with new family members. Thus, the investigation of gene-replication events can assist us in understanding the evolution of *Phyllostachys edulis*. Gene-duplication events can be divided into three categories: whole-genome duplication events, segmental duplication, and tandem duplication [22]. In our research, the main duplication event of PheNAP1 gene was whole-genome duplication, followed by segmental duplication, and no tandem replication events occurred. We used Ka/Ks to check whether there were evolutionary pressure on the evolution of PheNAP1 [23]. The outcomes showed that the Ka/Ks values of each PheNAP1 homologous, duplicated gene pair were all less than 1, demonstrating that the PheNAP1 gene pairs had undergone strong purification selection during the evolution process and would be functionally conserved due to negative selection. Synteny analysis of PheNAP1 showed that *Phyllostachys edulis* is collinear with the monocotyledons *Oryza sativa* and *Zea mays*, yet not with the dicotyledon *Arabidopsis thaliana*, which is consistent with the evolutionary relationship between monocotyledons and dicotyledons.

Genes have different expression patterns in different tissues and organs, and thus they have different biological functions [24]. PheNAP1 was expressed in all organs, indicating that it played a role in various growth stages and tissues of *Phyllostachys edulis*. Previous studies have shown that PheNAP1 also plays a crucial part in primitive cells with strong meristem ability, such as the shoot apical meristem region and the young internode of moso bamboo (Figure S3) [25]. NAP1 is also involved in plant somatic homologous recombination [9], plant root growth [7], plant flowering, and other processes, suggesting that NAP1 may be extensively involved in plant growth and development. The high expression level in the shoots, especially in 0.2 m–0.5 m shoots, indicates that PheNAP1 may also be involved in the fast growth of *Phyllostachys edulis*. Interestingly, PheNAP1:1 can rescue the early-flowering phenotype of *nrp1-1 nrp2-2*, and the expression level of *BSU1* showed a negative correlation with *PheNAP1:1*. Considering *BSU1* is the inhibitor of *FLC*, the key repressor of flowering [26], PheNAP1 is assumed to engage in regulating the flowering of moso bamboo, and its high expression in flower florets adds more proof to it.

Histone methylation, phosphorylation, and acetylation are important epigenetic markers that are critical for regulating chromatin remodeling processes, leading to a better understanding of the epigenetic regulation of gene activation and silencing. Histone modification results showed that PheNAP1:1 could bind to the classically modified histones, and further, it may affect the fast growth and flowering of *Phyllostachys edulis*. Studies have found that H3S10P begins in the early stage of cell division, reaches its peak at the middle stage of division, and declines at the end of division as the overall phosphorylation level decreases [27]. It has been found in studies on maize that H3S10P occurs in the prophase of mitosis and meiosis [28]. Histone methylation, one of the most complex modifications, occurs on arginine and lysine residues of H3 and H4, which activate or inhibit gene transcription. For example, H3K4me2/3, H3K36me1/3, H3K79me1/2, and H4K20me1 are associated with transcriptional activation, while H3K9me2/3, H3K27me2/3, H3K79me3, and H4K20me3 are associated with transcriptional inhibition. Histone acetylation can also affect the activation and inhibition of gene transcription.

It is reported that gibberellin (GA) is one vital factor in the rapid growth of moso bamboo [29], and the relationship between PheNAP1 and DELLA, the key gene in the GA-related pathway, could be surveyed. Although we observed the negative relationship between *PheNAP1* and *BSU1*, the genes of this regulatory pathway needed to be further clarified. Thus, although the histone chaperone PheNAP1 may closely relate to the two important development processes of moso bamboo, rapid growth and flowering, more evidence is still needed.

## 4. Materials and Methods

### 4.1. Plant Materials

The seeds of *Phyllostachys edulis* used in this study were purchased from Guilin, Guangxi province. The 1-meter-long shoots were collected from the Bamboo Botanical Garden of Fujian Agricultural Forestry University (Fujian Province of China, E119°140; N26°050) in April 2020. All of the *Arabidopsis thaliana* lines used have a Columbia-0 background. The mutant line *nrp1-1* is Salk_117793, and *nrp2-2* is Salk_205276. The hybrid material *nrp1-1 nrp2-2* double mutant was obtained by crossing *nrp1-1* and *nrp2-2*. For seed planting, plants were grown under standard long-day conditions (22 °C, 16 h light/8 h dark) in a greenhouse. For resistant plant screening, seeds were planted on MS medium (MS, 1% sucrose, MES hydrate, pH 5.7, and 0.7% agar) after the surface was sterilized. The plants were kept at 4 °C for 2–3 days in the dark and then grown under standard long-day conditions.

### 4.2. Data Resources

The chromosome-level reference genome and annotation file of moso bamboo were retrieved from the (*Phyllostachys edulis*) GigaScience Database (http://gigadb.org/dataset/100498, accessed on 23 December 2021) [30]. Genomic data of *Arabidopsis thaliana* was downloaded from TAIR (https://www.arabidopsis.org/, accessed on 23 December 2021); that of *Oryza sativa* from RGAP (http://rice.uga.edu/, accessed on 23 December 2021, version 7.0), and that of Zea mays was downloaded from the maize genetics and genomics database (https://www.maizegdb.org/download, accessed on 23 December 2021). RNA-seq data was downloaded from https://github.com/StefanReuscher/youngBambooShootsRNAseq (accessed on 16 September 2021) [25]. Bioproject ERP001341 was deposited in EMBL (https://www.ebi.ac.uk, accessed on 16 September 2021) [15] and NCBI (https://www.ncbi.nlm.nih.gov, accessed on 16 September 2021) under accession PRJNA414226 [31], PRJNA496390 [20]. BS-Seq data have been deposited at NCBI (accessed on 16 September 2021) under accession number PRJNA503816 [20].

### 4.3. Identification of NAP1 Family Genes in Phyllostachys edulis

In order to identify the NAP1 family proteins of *Phyllostachys edulis*, we used the NAP1 protein sequence of *Arabidopsis thaliana* as a template for BLASTP. Next, Hidden Markov models (HMMs) [32] were constructed using the NAP domain, and the specific NAP1 family sequence was searched. We used the ExPASy ProtParam tool [33] (https://web.expasy.org/protparam/, accessed on 24 December 2021) to analyze the physical and chemical properties, including the molecular weight, isoelectric point, etc. We used the Plant-mPLoc tool [34] (http://www.csbio.sjtu.edu.cn/bioinf/plant-multi/, accessed on 24 December 2021) for subcellular localization analysis.

### 4.4. Analysis of Phylogeny, Conserved Motif and Gene Structure

The intron–exon distribution of NAP1 was analyzed by TB tools [35], using the genome annotation files and gene ID of *Phyllostachys edulis*, and the gene structure was mapped. The MEME online analysis tool [36] (https://meme-suite.org/meme/, accessed on 24 December 2021) was used to analyze the conserved motifs of the NAP1 protein. The maximum basic number was set to 15, and the rest were set as default parameters. We used NCBI CDD (https://www.ncbi.nlm.nih.gov/Structure/bwrpsb/bwrpsb.cgi, accessed on 24 December 2021) for protein structure domain analysis. MAGAX [37] was used to construct the phylogenetic tree, and the neighbor-joining (NJ) generation algorithm was adopted. Parameters were set as follows: “Poisson Model”, “Complete deletion”, Bootstrap = 1000 times.

### 4.5. Chromosome Location and Synteny Analysis

The location information of the NAP1 gene was obtained from the annotated file of *Phyllostachys edulis*, and the chromosome location maps were plotted using Map Gene2 Chromosome V2 (http://mg2c.iask.in/mg2c_v2.0/, accessed on 24 December 2021). MCScanX [38] was used to analyze the gene duplication events within the genome of *Phyllostachys edulis* and the synteny relationship between *Phyllostachys edulis* and other species. The default parameters were used for all parameters, and TBtools was used to visualize the results.

### 4.6. Estimation of Ka/Ks Ratios

Ka and Ks rates between PheNAP1 gene pairs were calculated using KaKs Calculator 2.0 [39].

### 4.7. Cis-Acting Element Analysis

The sequence 3000bp upstream of the NAP1 gene sequence was extracted using the PlantCARE [40] analysis tool (http://bioinformatics.psb.ugent.be/webtools/plantcare/html/, accessed on 28 December 2021) for cis element analysis, and TBtools was used to visualize results.

### 4.8. Expression Analysis from RNA-seq Data

From the NCBI gene expression profile database (http://www.ncbi.nlm.nih.gov/geo/, accessed on 16 September 2021), we downloaded the transcriptome data and the original SRA data onto FASTQ to filter out low-quality reads. Hisat 2 software [41] was used to conjugate it with the default parameters set to the second edition of the moso bamboo genome reference sequence, and Samtools [42] was used to transform them. The combined SAM was sorted into a BAM file. We calculated the standardized gene expression fragments per kilobase of transcript per million mapped (FPKM) using Stringtie [43,44].

### 4.9. RNA Extraction and Expression Analysis

Total RNA were extracted from different tissues (roots, leaves, and stems) of 3-week-old moso bamboo and 1-meter-long shoots using TRizol and an RNeasy^®^ Plant Mini Kit (QIAGEN, Hilden, Germany, Cat. No. 74904). Sample cDNA were synthesized via a PrimeScript™ RT Reagent Kit with gDNA Eraser (TaKaRa, Kusatsu City, Japan, Cat. No. RR047A) using 2mg of RNA. RT-qPCR primers were designed using Primer3 Plus (https://www.primer3plus.com/index.html, accessed on 5 March 2022) and listed in Table S3. We used proprietary software with Quant Studio 6 (Life Technologies, Carlsbad, CA, USA) and GoTaq^®^ qPCR Master Mix (PROMEGA, Madison, WI, USA, Cat. No. A6002) for RT-qPCR reactions. The reaction system was as follows: 2× MasterMix 10 µL, Primer F 1 µL (10 μM), Primer R 1 µL (10 μM), cDNA 1 µL, and nuclease-free water up to 20 µL. The reaction procedure was as follows: pre-denaturation at 95 °C for 30 s; denaturation at 95 °C for 15 s, annealing at 60 °C for 30 s, elongation at 72 °C for 1 min/Kb, 40 cycles, The melt curve was analyzed immediately after the reaction was completed, and the procedure was as follows: 95 °C for 15 s; 60 °C for 1 min; 95 °C for 15 s. Relative expression was calculated by the 2^−ΔΔCT^ method. For all quantitative analyses, we performed three biological replicates and three technical replicates. An unpaired *t*-test was used to analyze the significance of each tissue of the moso bamboo. Genes were considered significantly deregulated when fold change was ≥1.5 and *p*-value < 0.05.

### 4.10. Histone Modification Analysis

The purified protein was tested for histone modification using a MODified Histone Peptide Array (Active Motif, Carlsbad, California, CA, USA, Cat. No. 13005). After the detection was complete, we used Array Analyze software.

### 4.11. Immunofluorescence

We placed 4–6 leaves into Buffer 1 (1M Tris-HCl pH 7.5, 4% paraformaldehyde), vacuumed for 2 min, and put it on ice for 30 min. Next, we cleaned the sample using Buffer 1; after that, we added LB01 Buffer (1M Tris-Hcl pH 7.5, Spermine-4HCl, KCl, NaCl, NaEDTA, and Triton X-100). The sample was shredded, and the nucleus was released. Then, Nylon Net Filters (Millipore, Burlington, MA, USA Cat. No. NY4102500) were used to filter the sample, and filtration solution was added to the slides and dropped into the sorting buffer (1M Tris-HCl pH 7.5, MgCl, KCl, Tween™ 20, and sucrose) and left to dry at room temperature. We then put the slides into Buffer 2 (PBS, 4% paraformaldehyde) and incubated this for 20 min at room temperature. After several cleanings, we added SuperBlock™ Blocking Buffer (Thermo Scientific™, Waltham, MA, USA, Cat. No. 37515) and incubated them at 37 °C for 30 min in the dark. Next, we added primary and secondary antibodies, and then we added Vectashield with DAPI. We used a Zeiss LSM880 Airyscan (Zeiss, Oberkochen, Germany) for analysis.

## 5. Conclusions

This is the first identification and characterization of NAP1, the main chaperone of histone H2A–H2B in *Phyllostachys edulis*. A total of 12 PheNAP1 members were recognized, which can be divided into two subfamilies, NRP and NAP. According to the gene structure and conserved domain, the PheNAP1 family has been exceptionally conserved through evolution. RNA-seq data and RT-qPCR results showed that PheNAP1 expression was tissue-specific and highly expressed in tissues with strong meristem capacity, such as fast-growing shoots. PheNAP1 also affects the expression of key genes in the flowering pathway, such as *BSU1*. Taken together, histone chaperone and PheNAP1 may be engaged in the rapid growth and flowering processes in *Phyllostachys edulis*. Thus, epigenetic regulation is a vital part of the development of bamboo plants. Our results will lay a foundation for further studies of chromatin function.

## Data Availability

Not applicable.

## Supplementary Materials

The supporting information can be downloaded at: https://www.mdpi.com/article/10.3390/ijms23126491/s1.
