# Supplementary material for: Genome-Wide Identification of NAP1 and Function Analysis in Moso Bamboo (Phyllostachys edulis)
Source: Int J Mol Sci. 2022 Jun 10;23(12):6491. doi: 10.3390/ijms23126491 (PMC9223780; doi:10.3390/ijms23126491)
Supplement: Supplementary file 1 [file ijms-23-06491-s001.zip › ijms-1754171-supplementary.pdf]

International Journal of *Molecular Sciences*

Article

Genome-Wide Identification of NAP1 and Function Analysis in Moso Bamboo (*Phyllostachys edulis*)

Yaxing Zhang [1], Jun Zhang [2], Deming Yang [3], Yandong Jin [1], Xuqing Liu [1], Zeyu Zhang [1], Lianfeng Gu [1,*] and Hangxiao Zhang [1,*]

[1] College of Forestry, Basic Forestry and Proteomics Research Center, Fujian Agriculture and Forestry University, Fuzhou 350002, China; yaxinzhang0823@gmail.com (Y.Z.); 1200421007@fafu.edu.cn (Y.J.); fafuxuqing@163.com (X.L.); zeyubio0212@gmail.com (Z.Z.)
[2] College of Life Science, Basic Forestry and Proteomics Research Center, Fujian Agriculture and Forestry University, Fuzhou 350002, China; jessy_1999@163.com
[3] College of Forestry, Fujian Agriculture and Forestry University, Fuzhou 350002, China; demingyang0721@gmail.com
* Correspondence: lfgu@fafu.edu.cn (L.G.); zhanghx@fafu.edu.cn (H.Z.)

Supplementary Materials

Citation: Zhang, Y.; Zhang, J.; Yang, D.; Jin, Y.; Liu, X.; Zhang, Z.; Gu, L.; Zhang, H. Genome-Wide Identification of NAP1 and Function Analysis in Moso Bamboo (*Phyllostachys edulis*). *Int. J. Mol. Sci.* **2022**, *23*, 6491. https://doi.org/10.3390/ijms23126491

Academic Editor: Abinaya Manivannan

Received: 18 May 2022
Accepted: 8 June 2022
Published: date: 10 June 2022

Publisher's Note: MDPI stays neutral with regard to jurisdictional claims in published maps and institutional affiliations.

Copyright: © 2022 by the authors. Submitted for possible open access publication under the terms and conditions of the Creative Commons Attribution (CC BY) license (https://creativecommons.org/licenses/by/4.0/).

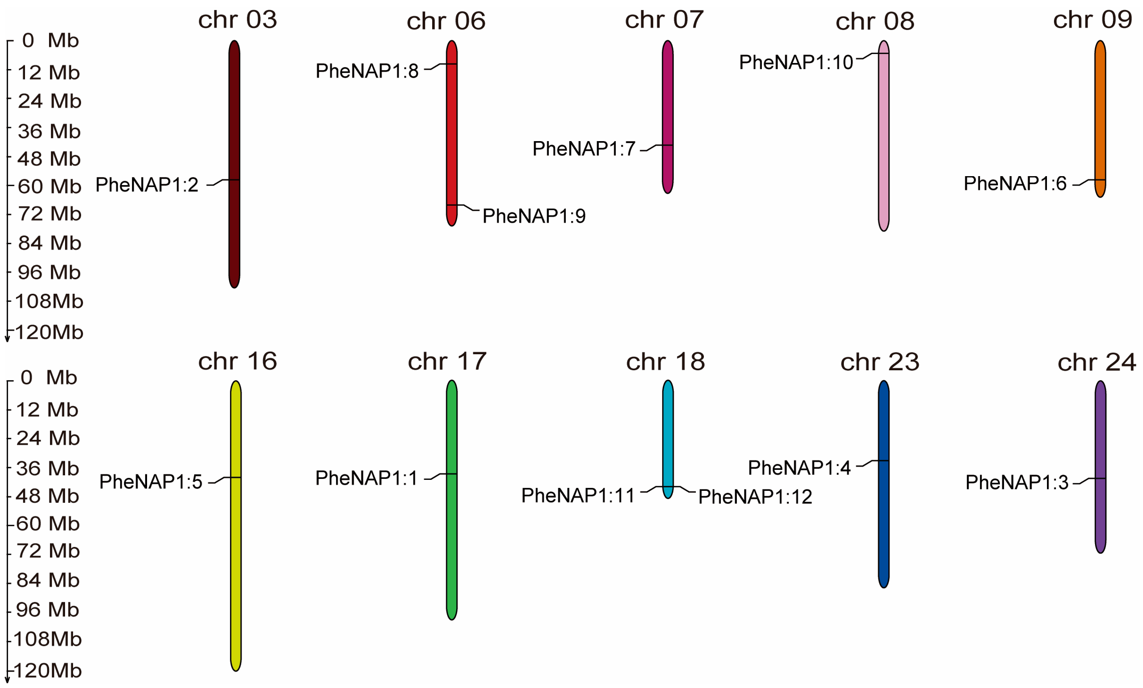

Figure S1. Chromosome location a of NAP1 genes in Phyllostachys edulis. Locations of the PheNAP1 genes in chromosomes. Chromosome length is shown on the far left. Different chromosomes are shown in different colors, length indicates the size of the chromosome, and PheNAP1 is marked at approximate locations on the chromosome.

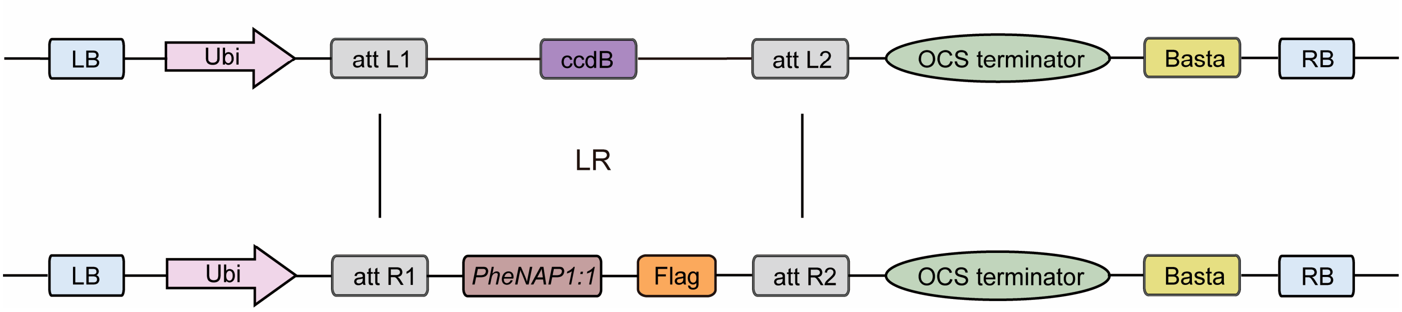

Figure S2. The construction model of PEG-Ubi-PheNAP1:1 expression vector for immunofluorescence. LB: left boundary of T-DNA; Ubi, maize ubiquitin promoter; Att L1, Att L2, Att R1, Att R2: homologous recombination sites; CcdB: lethal protein gene; Flag: protein detection label; OCS terminator: transcriptional termination signal; Basta: screening marker RB:right boundary of T-DNA.

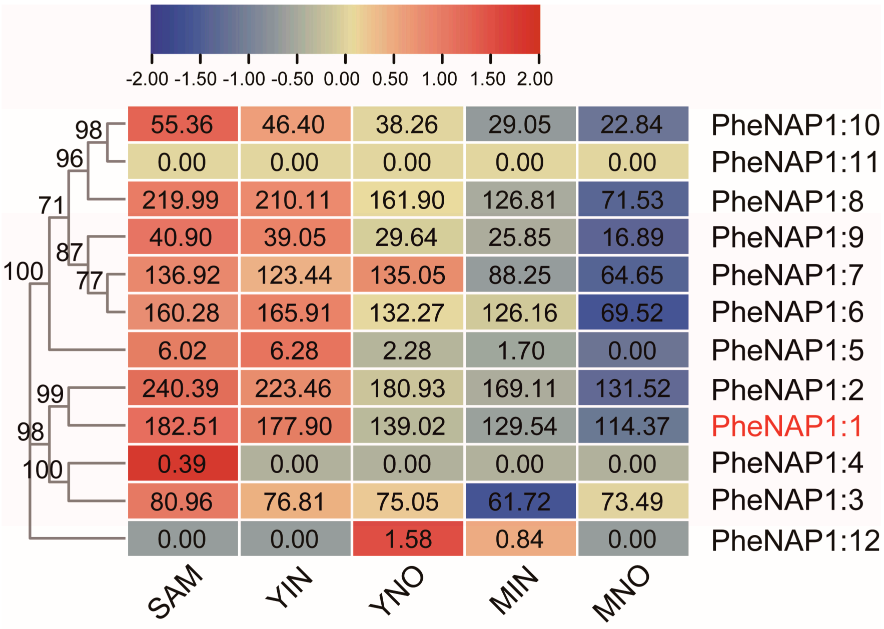

Figure S3. Expression analysis of PheNAP1 in different parts of shoot of *Phyllostachys edulis*. SAM: shoot apical meristem region ; YIN: young internode ; MIN : mature internode ; YNO : young node ; MNO: mature node.

Table S1. Characteristics of the NAP1 identified in *Phyllostachys edulis*.

Gene Name	Gene ID	Exon Count	Chr	CDS (bp)	Amino Acid (aa)	MW (kDa)	PI	Location
PheNAP1:1	PH02Gene 24995.t 1	10	17	756	251	28.57	4.24	Nucleus
PheNAP1:2	PH02Gene 44771.t 1	10	3	765	254	28.95	4.24	Nucleus
PheNAP1:3	PH02Gene 02215.t 1	10	24	765	254	29. 1	4.36	Nucleus
PheNAP1:4	PH02Gene 10055.t 1	7	23	612	203	23. 17	4.93	Nucleus
PheNAP1:5	PH02Gene 05059.t 1	11	16	1071	356	40.25	4.34	Nucleus
PheNAP1:6	PH02Gene 45207.t 1	11	9	1086	361	41.21	4.39	Nucleus
PheNAP1:7	PH02Gene 12888.t2	10	7	1176	391	44.36	4.34	Nucleus
PheNAP1:8	PH02Gene 19479.t 1	12	6	1137	378	42.98	4.32	Nucleus
PheNAP1:9	PH02Gene 42258.t 1	12	6	933	310	35.64	4.68	Chloroplast/ Mitochondrion/ Nucleus
PheNAP1:10	PH02Gene 25565.t 1	15	8	1488	495	56.72	5.73	Chloroplast
PheNAP1:11	PH02Gene 06799.t 1	4	18	276	91	10.48	5.07	Nucleus
PheNAP1:12	PH02Gene 06798.t 1	6	18	675	224	24.45	4.52	Chloroplast/ Nucleus

Table S2. Analysis of segmental duplication events of NAP1 gene pairs in *Phyllostachys edulis.*

Locus 1	Chromosome localization	Locus 2	Chromosome localization
PH02Gene05059.t1	Chr16:40653549–40658810	PH02Gene12888.t2	Chr7:43905498–43909478
PH02Gene05059.t1	Chr16:40653549–40658810	PH02Gene45207.t1	Chr9:58319518–58322973
PH02Gene24995.t1	Chr17:39439636–39445102	PH02Gene10055.t1	Chr23:33515135–33520455
PH02Gene24995.t1	Chr17:39439636–39445102	PH02Gene02215.t1	Chr24:40988176–40995165
PH02Gene24995.t1	Chr17:39439636–39445102	PH02Gene44771.t1	Chr3:58436448–58441153
PH02Gene10055.t1	Chr23:33515135–33520455	PH02Gene02215.t1	Chr24:40988176–40995165
PH02Gene10055.t1	Chr23:33515135–33520455	PH02Gene44771.t1	Chr3:58436448–58441153
PH02Gene02215.t1	Chr24:40988176–40995165	PH02Gene44771.t1	Chr3:58436448–58441153
PH02Gene19479.t1	Chr6:9678197–9682618	PH02Gene25565.t1	Chr8:5464900–5473744
PH02Gene12888.t2	Chr7:43905498–43909478	PH02Gene45207.t1	Chr9:58319518–58322973

Table S3. Primer list.

Primer Name	Sequence 5' to 3'
NAP1:1–QF	TTTGGCTGACAGCGTTTCTG
NAP1:1–QR	AATGGAGTAGCCCGACTTGAC
NAP1:2–QF	GGAATGAAGGAATGGGAACTGC
NAP1:2–QR	TGATCTCTGCCACCTCATCTTG
NAP1:3–QF	TCGAGAGGGTTAATGAGGAAGC
NAP1:3–QR	GCATAGGATGGCTAAGAAACGC
NAP1:4–QF	TCTCCTGCTCTCCATTGAGAAG
NAP1:4–QR	TCATTCCGCCGACCATAAAC
NAP1:5–QF	TGGGACGGAAATTGAATGGC
NAP1:5–QR	GAAGCTCTCGCAATCTTCAGTC
NAP1:6–QF	TGAGCCAAAGGGTTTCAAGC
NAP1:6–QR	TGGCTTTCTCTAGGATCGGTTC
NAP1:7–QF	TGTCAATGGTGTGGTTGAGG
NAP1:7–QR	TGCAGTAAGCCAGAAATCGG
NAP1:8–QF	GTCAATGCCCTAAAGGACAAGC
NAP1:8–QR	GCCTCAATCTCATCATGTTGGC
NAP1:9–QF	CAGCGGGGCCTTATTTCTTG
NAP1:9–QR	CATTGTTGCTTGCACTCTGC
NAP1:10–QF	TGAGGTTTTCTTGGCTGACC
NAP1:10–QR	ATCCTTTGCACGAAGTCTGG
NAP1:11–QF	GCTTCAGGGTCAAATGGAACATG
NAP1:11–QR	ACGGTAAACCAGGAAACAACATG
NAP1:12–QF	TGGAGTTGTGGAGGTTGATGG
NAP1:12–QR	ATCTGCATCTTTCCCCTCAGC
eIF 4a–F	AGTTCCGCCAATCAGCAATAC
eIF 4a–R	CCATAGGCAAGCAAGCATCC
